# Leaky ryanodine receptors in β-sarcoglycan deficient mice: a potential common defect in muscular dystrophy

**DOI:** 10.1186/2044-5040-2-9

**Published:** 2012-05-28

**Authors:** Daniel C Andersson, Albano C Meli, Steven Reiken, Matthew J Betzenhauser, Alisa Umanskaya, Takayuki Shiomi, Jeanine D’Armiento, Andrew R Marks

**Affiliations:** 1 Department of Physiology and Cellular Biophysics, Columbia University College of Physicians and Surgeons, New York, NY 10032, USA; 2 Current affiliation: Faculty of Medicine, Masaryk University, Brno, Czech Republic; 3 Department of Medicine, Columbia University College of Physicians and Surgeons, New York, NY 10032, USA; 4 Current affiliation: Department of Medicine, Karolinska Institutet, Stockholm, Sweden; 5 Clyde and Helen Wu Center for Molecular Cardiology, , New York, NY 10032, USA

**Keywords:** Muscular dystrophy, Ryanodine receptor, Calstabin1, Calcium

## Abstract

**Background:**

Disruption of the sarcolemma-associated dystrophin-glycoprotein complex underlies multiple forms of muscular dystrophy, including Duchenne muscular dystrophy and sarcoglycanopathies. A hallmark of these disorders is muscle weakness. In a murine model of Duchenne muscular dystrophy, *mdx* mice, cysteine-nitrosylation of the calcium release channel/ryanodine receptor type 1 (RyR1) on the skeletal muscle sarcoplasmic reticulum causes depletion of the stabilizing subunit calstabin1 (FKBP12) from the RyR1 macromolecular complex. This results in a sarcoplasmic reticular calcium leak via defective RyR1 channels. This pathological intracellular calcium leak contributes to reduced calcium release and decreased muscle force production. It is unknown whether RyR1 dysfunction occurs also in other muscular dystrophies.

**Methods:**

To test this we used a murine model of Limb-Girdle muscular dystrophy, deficient in β-sarcoglycan (Sgcb−/−).

**Results:**

Skeletal muscle RyR1 from Sgcb−/− deficient mice were oxidized, nitrosylated, and depleted of the stabilizing subunit calstabin1, which was associated with increased open probability of the RyR1 channels. Sgcb−/− deficient mice exhibited decreased muscle specific force and calcium transients, and displayed reduced exercise capacity. Treating Sgcb−/− mice with the RyR stabilizing compound S107 improved muscle specific force, calcium transients, and exercise capacity. We have previously reported similar findings in *mdx* mice, a murine model of Duchenne muscular dystrophy.

**Conclusions:**

Our data suggest that leaky RyR1 channels may underlie multiple forms of muscular dystrophy linked to mutations in genes encoding components of the dystrophin-glycoprotein complex. A common underlying abnormality in calcium handling indicates that pharmacological targeting of dysfunctional RyR1 could be a novel therapeutic approach to improve muscle function in Limb-Girdle and Duchenne muscular dystrophies.

## Background

Muscular dystrophies (MD) comprise a group of inherited disorders affecting striated muscles that are characterized by progressive weakness and muscle degeneration. The dystrophin-glycoprotein complex (DGC) is a macromolecular structure of membrane-associated proteins that includes dystrophin and the sarcoglycan proteins (α-, β-, δ-, and γ-sarcoglycan), which maintain fiber integrity and protect from contraction-induced muscle damage [1,2]. Mutation-induced disruption of sarcoglycan proteins leads to limb-girdle muscular dystrophy (LGMD) [3-5]. A null mutation in one of the sarcoglycans results in loss of the whole sarcoglycan complex but not of dystrophin [4,6]. However mutations in dystrophin, which cause the most common form of muscular dystrophy, Duchenne muscular dystrophy (DMD), also lead to loss of the sarcoglycans [7]. This points to the loss of sarcoglycans as the central upstream event in muscular dystrophies. Disruption of the DGC is associated with oxidative stress, activation of Ca^2+^-dependent neutral proteases (calpains) [8], mitochondrial Ca^2+^ overload, and apoptosis [9,10]. Moreover, pathological Ca^2+^ signaling has been attributed to MDs [11-17].

Skeletal muscle contraction is regulated by a process known as excitation-contraction (E-C) coupling. A critical feature of this process is the release of Ca^2+^ from the sarcoplasmic reticulum (SR) via the intracellular Ca^2+^ release channel/ryanodine receptor type 1 (RyR1). To initiate E-C coupling, depolarization of the cell membrane activates L-type calcium channels (Ca_v_1.1) on the transverse tubule, which then activates RyR1 through the direct interaction between the two ion channels, causing release of Ca^2+^ from the SR into the cytoplasm. The increase in Ca^2+^ enables the actin-myosin cross-bridge formation and sarcomere shortening that results in muscle contraction [18].

RyR1 is a macromolecular complex with associated regulatory proteins including kinases, phosphatases, and the peptidyl-propyl-*cis-trans*-isomerase FK506 binding protein 12 (FKBP12, also known as calstabin1). Calstabin1 binds to RyR1 and stabilizes the closed state of the channel, thereby preventing a potentially pathological Ca^2+^ leakage from the SR [19]. RyR1 has multiple cysteine residues that can be S-nitrosylated and S-glutathionylated at physiological pH [20]. These modifications can destabilize the closed state of the RyR1, which results in a pathological cytoplasmic Ca^2+^ ‘leak’ [21]. The RyR1 is, moreover, susceptible to oxidation-dependent modifications and we have recently shown that SR Ca^2+^ ‘leak’ contributes to age-dependent muscle weakness [22]. Furthermore, inhibition of this intracellular Ca^2+^ leak with a novel drug that stabilizes the RyR (S107) [22,23] reduces SR Ca^2+^ leak and improves muscle function in aged mice [22] and in the *mdx* mouse model of DMD [23].

In the present study we show that β-sarcoglycan-deficient mice (Sgcb−/− mice; an established murine model of LGMD) [3], display RyR1 phosphorylation, S-nitrosylation and oxidation, Ca^2+^ leak through RyR1, reduced tetanic Ca^2+^, and specific force in isolated fast twitch EDL muscles. Treatment with S107 reduced the Ca^2+^ leak, increased muscle Ca^2+^ release, force production, and improved voluntary exercise capacity in Sgcb−/− mice. Disruption of the DGC leads to a common molecular pathophysiological mechanism in both DMD and LGMD that involves maladaptations of the RyR1 and Ca^2+^ leak. Furthermore, this disease phenotype is likely to respond to therapy with a Ca^2+^ leak-reducing compounds and thus presents new pharmaceutical strategies in treating muscular dystrophies.

## Methods

### Animals

Homozygous β-sarcoglycan deficient mice (Strain: B6.129-*Sgcb*^tm1Kcam^/1 J; in this article referred to as Sgcb−/−) were obtained from The Jackson Laboratory (Bar Harbor, ME, USA) [3,24]. The Sgcb−/− mice were backcrossed for several generations into C57Bl/6 background and aged-matched C57Bl/6 mice were used as controls. All experiments with animals were approved by Columbia University’s Institutional Animal Care and Use Committee.

### Voluntary exercise and S107 treatment

At the beginning of each experiment mice were transferred to individual cages equipped with running wheels and exercise was recorded using a data acquisition system (Respironics). The mice were acclimated to the running wheels for 7 to 9 days and were randomized into two treatment groups. The first group received S107 (25 mg/100 mL) in the drinking water and the second group received water only. S107 (S107-HCl, FW 245.77) was synthesized as previously described [25-27]. The structure and purity of S107 were confirmed by NMR, MS, and elemental analysis [25]. The specificity of S107 was assessed against a panel of >250 channels, receptors, phosphatases, and kinases [25]. Mice drank approximately 9 mL/day (water bottle and body weight were recorded to monitor consumption) for a daily dose of S107 of approximately 1.5 mg. There was no difference in daily water consumption between the treatment groups (mean ± SEM: control, 9.9 ± 0.6 mL, S107, 9.3 ± 0.9 mL; *n* = 5, *P* = NS). Mice were sacrificed using CO_2_ followed by cervical dislocation and muscles were harvested for functional and biochemical analyses. Investigators performing all aspects of the studies were blinded to the treatment groups.

### Muscle function

Extensor digitorum longus (EDL) muscles were dissected from hind limbs. Stainless steel hooks were tied to the tendons of the muscles using nylon sutures and the muscles were mounted between a force transducer (Harvard Apparatus) and an adjustable hook. The muscles were immersed in a stimulation chamber containing O_2_/CO_2_ (95/5%) bubbled Tyrode solution (in mM: NaCl 121, KCl 5.0, CaCl_2_ 1.8,3 MgCl_2_ 0.5, NaH_2_PO_4_ 0.4, NaHCO_3_ 24, EDTA 0.1, glucose 5.5). Muscles were stimulated to contract using an electrical field between two platinum electrodes (Aurora Scientific). At the start of each experiment the muscle length (L_0_) was adjusted to yield the maximum force. The force-frequency relationships were determined by triggering contraction using incremental stimulation frequencies (EDL: 0.5 ms pulses at 2 to 150 Hz for 350 ms at supra-threshold voltage). The muscles were allowed to rest between every force-frequency stimulation for >1 min. At the end of the force measurement, the L_0_ and weight of the muscles were measured and the muscles were snap frozen in liquid N_2_. To quantify the specific force, the absolute force was normalized to the muscle cross-sectional area, calculated as the muscle weight divided by the length using a muscle density constant of 1.056 kg*m^-3^[28].

### Muscle fatigue protocol

After force-frequency measurements, the EDL muscle was fatigued. The fatigue protocol for the EDL muscle consisted of 50 tetanic contractions (70 Hz, 350 ms duration) given at 2-s intervals.

### RyR1 immunoprecipitation and immunoblotting

EDLs were isotonically lysed in 0.5 mL of a buffer containing 50 mM Tris–HCl (pH 7.4), 150 mM NaCl, 20 mM NaF, 1.0 mM Na_3_VO_4_, and protease inhibitors. An anti-RyR antibody (4 μg 5029 Ab) was used to immunoprecipitate RyR1 from 250 μg of tissue homogenate. The samples were incubated with the antibody in 0.5 mL of a modified RIPA buffer (50 mM Tris–HCl pH 7.4, 0.9% NaCl, 5.0 mM NaF, 1.0 mM Na_3_VO_4_, 1% Triton-X100, and protease inhibitors) for 1 h at 4°C. The immune complexes were incubated with protein A Sepharose beads (Sigma, St Louis, MO, USA) at 4°C for 1 h and the beads were washed three times with buffer. Proteins were separated on SDS-PAGE gels (6% for RyR1, 15% for calstabin1) and transferred onto nitrocellulose membranes for 1 h at 200 mA (SemiDry transfer blot, Bio-Rad). After incubation with blocking solution (LICOR Biosciences, Lincoln, NE, USA) to prevent non-specific antibody binding, immunoblots were developed with anti-RyR (Affinity Bioreagents, Bolder, CO, USA; 1:2,000), and anti-Cys-NO antibody (Sigma, St Louis, MO, USA; 1:2,000), or an anti-calstabin antibody (1:2,500). To determine channel oxidation the carbonyl groups on the protein side chains were derivatized to 2,4- dinitrophenylhydrazone (DNP-hydrazone) by reaction with 2,4 dinitrophenylhydrazine (DNPH). The DNP signal on RyR1 was detected by immunoblotting with an anti-DNP antibody. All immunoblots were developed and quantified using the Odyssey Infrared Imaging System (LICOR Biosystems, Lincoln, NE, USA) and infrared-labeled secondary antibodies.

### SR vesicle preparation

About 100 mg of isolated mouse EDL muscle was homogenized using a tissue mizer (Fisher Scientific) at the highest speed for 1 min with two volumes of: 20 mM Tris-maleate (pH 7.4), 1 mM EDTA, and protease inhibitors (Roche). Homogenate was centrifuged at 4,000 g for 15 min at 4°C and the following supernatant was centrifuged at 40,000 g for 30 min at 4°C. The final pellet, containing the SR fractions, was resuspended and aliquoted using the following solution: 250 mM sucrose, 10 mM MOPS (pH 7.4), 1 mM EDTA, and protease inhibitors. Samples were frozen in liquid nitrogen and stored at −80°C.

### Single-channel recordings

SR vesicles containing RyR1 were fused to planar lipid bilayers formed by painting a lipid mixture of phosphatidylethanolamine and phosphatidylcholine (Avanti Polar Lipids) in a 3:1 ratio in decane; across a 200-μm hole in polysulfonate cups (Warner Instruments) separating two chambers. The *trans* chamber (1.0 mL), representing the intra-SR (luminal) compartment, was connected to the head stage input of a bilayer voltage clamp amplifier. The *cis* chamber (1.0 mL), representing the cytoplasmic compartment, was held at virtual ground. Solutions used were the following: (in mM): 1 mM EGTA, 250/125 mM Hepes/Tris, 50 mM KCl, 0.54 mM CaCl_2_, pH 7.35 as *cis* solution, and 53 mM Ca(OH)_2_, 50 mM KCl, 250 mM Hepes, pH 7.35 as *trans* solution. The concentration of free Ca^2+^ in the *cis* chamber was calculated with WinMaxC program (version 2.50; http://www.stanford.edu/~cpatton/maxc.html). SR vesicles were added to the *cis* side and fusion with the lipid bilayer was induced by making the *cis* side hyperosmotic by the addition of 400 to 500 mM KCl. After the appearance of potassium and chloride channels, the *cis* side was perfused with the *cis* solution. Single-channel currents were recorded at 0 mV by using a Bilayer Clamp BC-525 C (Warner Instruments), filtered at 1 kHz using a Low-Pass Bessel Filter 8 Pole (Warner Instruments), and digitized at 4 kHz. To confirm RyR identity, 5 μM of ryanodine and/or 20 μM of ruthenium red were added at the end of each experiment. All experiments were performed at room temperature (23°C). Po was determined over 2 min of continuous recording using the method of 50% threshold analysis [29]. The recordings were analyzed by using Clampfit 10.1 (Molecular Devices) and Sigma Plot software (ver. 10.0, Systat Software), and Prism (ver.5.0, GraphPad).

### Ca^2+^ imaging in FDB muscle fibers

Single FDB fibers were obtained by enzymatic dissociation as previously described [30]. FDB muscles from both hind limbs were incubated for approximately 2 h at 37°C in approximately 4 mL Dulbecco’s Modified Eagles Medium (DMEM) containing 0.3% collagenase 1 (Sigma) and 10% fetal bovine serum. The muscles were transferred to a culture dish containing fresh DMEM (approximately 4 mL) and gently triturated using a 1,000 μL pipette until the muscles were dissociated. The cell suspension was stored in an incubator at 37°C/5% CO_2_ until the start of the experiment. FDB fibers were loaded with the fluorescent Ca^2+^ indicator Fluo-4 AM (5 μM, Invitrogen/Molecular probes) for 15 min in RT. The cells were allowed to attach to a laminin-coated glass cover slip that formed the bottom of a perfusion chamber. The cells were then superfused with tyrode solution (in mM: NaCl 121, KCl 5.0, CaCl_2_ 1.8, MgCl_2_ 0.5, NaH_2_PO_4_ 0.4, NaHCO_3_ 24, EDTA 0.1, glucose 5.5; bubbled with O_2_/CO_2_ (95/5%)). The fibers were triggered to tetanic contraction using electrical field stimulation (pulses of 0.5 ms at supra-threshold voltage, at 70 Hz for 350 ms) and Fluo-4 fluorescence was monitored using confocal microscopy (Zeiss LSM 5 Live, 40x oil immersion lens, excitation wavelength was 488 nm and the emitted fluorescence was recorded between 495 nm and 525 nm) in linescan mode. Only cells that were firmly attached to the glass bottom dish throughout the tetanic stimulation were included in the analysis. After subtraction of background fluorescence, the change in fluorescent signal during the tetanus (peak–resting (ΔF)) was divided by the resting signal (ΔF/F_0_). All experiments were performed at RT (approximately 20°C). The investigators were blinded to the genotype and treatment of subjects.

### Histology

**Figure 1:**
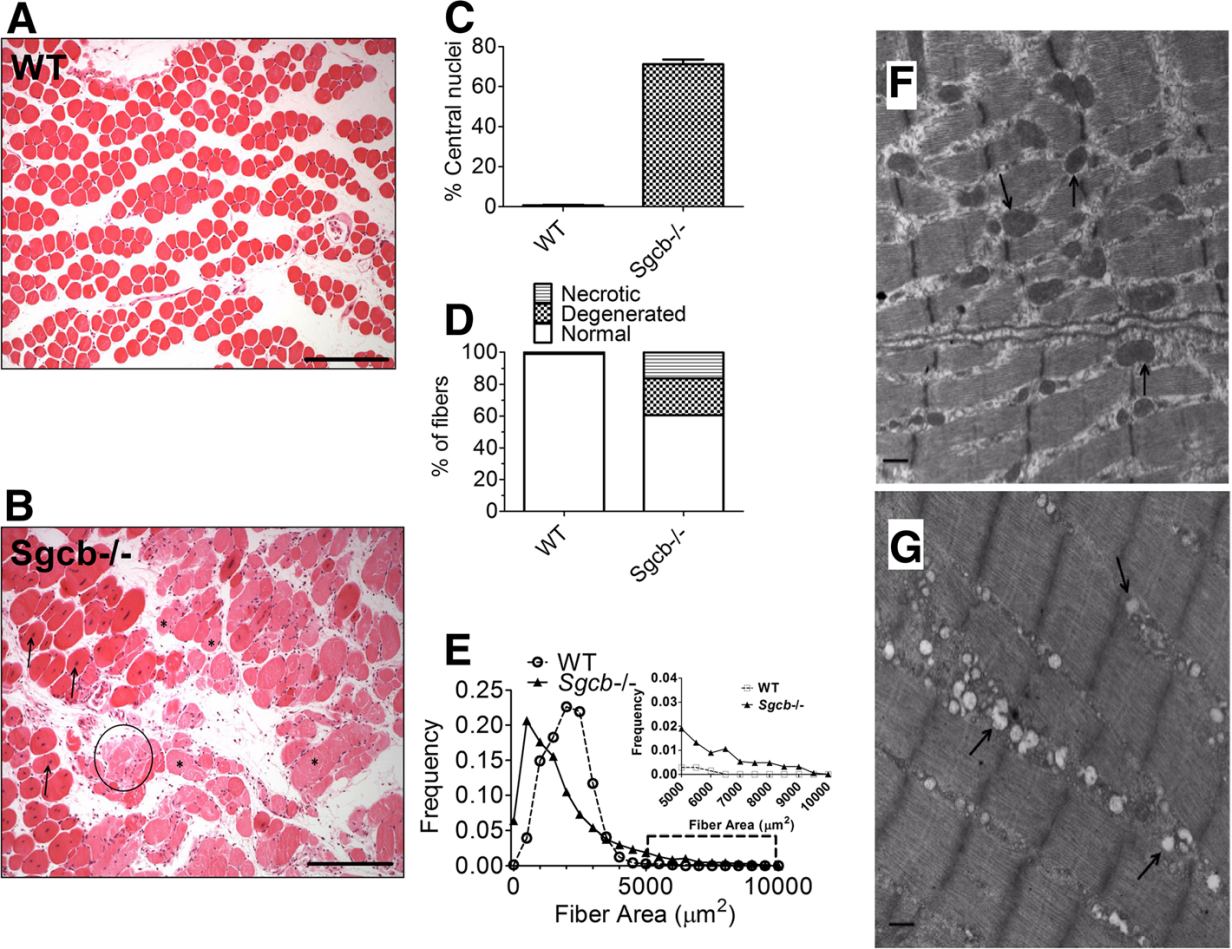
**EDL muscles from β-sarcoglycan deficient mice exhibit dystrophic morphology and abnormal mitochondrial morphology.** (**A**, **B**) EDL muscle cross-sections from wild-type (WT) and β-sarcoglycan mice stained with hematoxylin and eosin. (**C**) Percentage of fibers with the nucleus localized in the center (average ± SEM). (**D**) Percentages of normal, degenerated (weak eosin staining, examples indicated by asterisk) and necrotic (loss of eosin stain and swollen fiber, example indicated by a circle) muscle fibers. (**E**) Fiber size was more variable in Sgcb−/− EDL. This is indicated by the difference in the frequency distribution of fiber cross-sectional area. The inset in (E) is an expansion of the region indicated by the dashed rectangle in the main graph. Data were obtained from four mice and > 600 fibers in each group. The scale bar in images (A) and (B) indicate 250 μm. Representative electron microscopy images of EDL muscle from (**F**) WT and (**G**) Sgcb−/− mice. Arrows indicate normal mitochondria (F) or mitochondria with abnormal morphology, including low cristae density (G). Images from 11 fibers and two mice in each group were investigated under blinded conditions. The sample is magnified at × 25,000. Scale bar indicates 500 nm.

The EDL samples were fixed with formalin, embedded in paraffin wax, and sliced at 5 μm thickness. The sections were deparaffinized, stained with hematoxylin and eosin (H&E staining, Sigma-Aldrich Co., St Louis, MO, USA) and observed using light microscopy. The images were captured using a SPOT RT slider camera (Diagnostic Instruments Inc., Sterling Heights, MI, USA). For morphological analysis, images were taken randomly from each section using a computer controlled motorized stage. Then each image was analyzed by Image-Pro Plus software (Media Cybernetics, Inc., Bethesda, MD, USA). The judgment of qualitative parameters was performed by a clinical pathologist blinded to the mouse genotype. Degenerated fibers were defined as having weaker eosin staining, which was furthermore confirmed by weaker Gomori Trichrome staining (examples weak eosin staining is indicated by asterisks in Figure 1B). Necrotic fibers were defined as a swollen/degraded fiber with loss of eosin stain, with or without inflammatory cell infiltration (example is indicated by a circle in Figure 1B).

### Transmission electron microscopy

EDL muscles were fixed in 2.5% glutaraldehyde in 0.1 M Sorenson’s buffer (PH 7.2) followed by 1 h of post-fixation with 1% OsO4 in Sorenson’s buffer. After dehydration the tissue samples were embedded in Lx-112 (Ladd Research Industries) and 60 nm sections were cut using an ultramicrotome (MT-7000). The sections were then stained with uranyl acetate and lead citrate and examined under an electron microscope (JEM-1200 EXII, JEOL) and images were taken using an ORCA-HR digital camera (Hamamatsu) and recorded with an AMT Image Capture Engine.

## Results and discussion

Muscular dystrophy is accompanied by abnormal muscle morphology, including fiber degeneration and focal necrosis, which are associated with an enhanced regenerative activity in the muscle [3,31-33]. To confirm the dystrophic phenotype in the Sgcb−/− mice, we examined histopathological changes in EDL muscles from β-sarcoglycan-deficient mice compared to WT (Figure 1A-E). A majority (approximately 75%) of the muscle fibers from Sgcb−/− mice displayed centrally localized nuclei as opposed to the subsarcolemmal nuclei that are normally found in the healthy WT muscle (Figure 1C). This finding is consistent with regenerative activity in the muscle and has previously been reported in β-, and δ-sarcoglycan-deficient muscle [3,32,33]. Moreover, the Sgcb−/− muscle displayed overt histopathological changes, with a high prevalence of degenerated and necrotic fibers and a larger variability in the muscle fiber size (Figure 1B, D, and E). These morphological changes are typical for muscular dystrophy [3,32,33]. Mitochondrial abnormalities have also been described in patients [34] and murine models [9,31] of muscular dystrophy. Accordingly, ultrastructural analysis of EDL muscles from Sgcb−/− mice revealed many fibers with abnormal mitochondrial morphology, such as swelling and loss of cristae structure (Figure 1F, G). However, the sarcomere ultrastructure appeared normal in the Sgcb−/− muscle fibers (Figure 1F, G).

A hallmark of muscular dystrophies is limb muscle weakness [7]. We used extensor digitorum longus (EDL) muscles from 4- to 6-month-old Sgcb−/− mice and aged-matched wild-type controls (WT) to examine muscle force production. Isolated EDL muscles were electrically stimulated to contract and force production was measured. EDL muscle from Sgcb−/− mice displayed reduced absolute force compared to WT (mean tetanic force at 70 Hz stimulation ± SEM: Sgcb−/−, 280 ± 24 mN, *vs*. WT, 420 ± 30 mN; *n* = 9 (Sgcb−/−), *n* = 6 (WT); *P* <0.01 (*t*-test)). When the force was normalized to muscle cross-sectional area (specific force) the Sgcb−/− EDL muscles exhibited reduced specific force (mean force at 70 Hz stimulation ± SEM: Sgcb−/−, 200 ± 20 kNm^-1^, *vs*. WT, 440 ± 30 kNm^-1^; *n* = 9 (Sgcb−/−), *n* = 6 (WT); *P* <0.01 (*T*-test)), indicating defective force generation that is independent of muscle size. A pathognomonic sign in LGMD is the presence of pseudo-hypertrophy. Indeed, the EDL muscle mass was increased in Sgcb−/− (Sgcb−/−, 18 ± 1 mg, *vs*. WT, 13 ± 0.4; *n* = 9 (Sgcb−/−), *n* = 6 (WT); *P* <0.001), as previously published [6,35].

**Figure 2:**
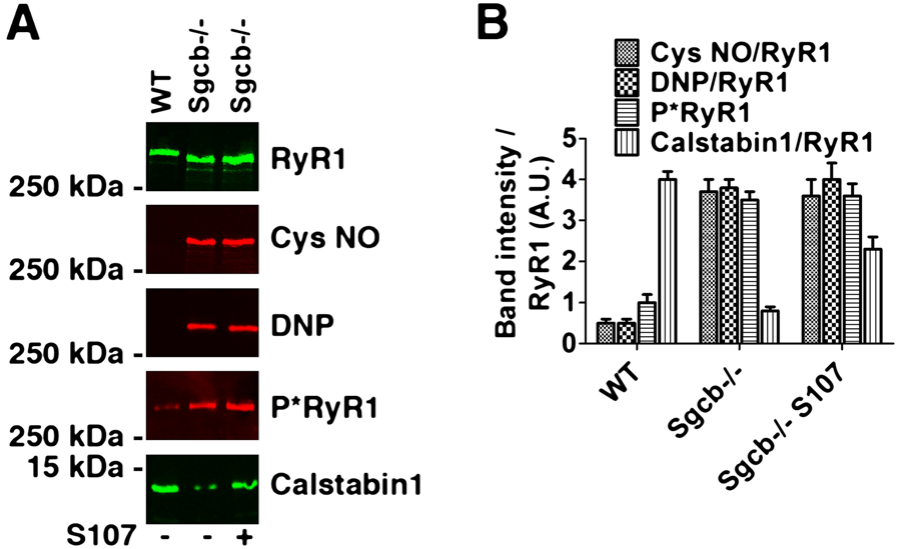
**RyR1 in β-sarcoglycan deficient muscle is cysteine-nitrosylated, oxidized, and depleted of calstabin1.** (**A**) Representative immunoblot of immunoprecipitated RyR1 from wild-type (WT) and β-sarcoglycan deficient (Sgcb−/−) EDL muscles. Antibodies against RyR1-S2844 phosphorylation (P*RyR1), cysteine-nitrosylated (Cys NO) proteins, calstabin1, and the protein oxidation marker 2,4- dinitrophenylhydrazone (DNP) was used. The muscle from a mouse treated with S107 is marked (+). (**B**) Bar graph showing average band intensities normalized to RyR1 expression (mean ± SEM, *n* = 3 for all groups).

To determine whether the observed reductions in muscle specific force were associated with remodeling of the RyR1 macromolecular complex, RyR1 were immunoprecipitated and immunoblotted to assay for post-translational modifications [23]. Skeletal muscle RyR1 channels from Sgcb−/− mice exhibited significantly increased phosphorylation, oxidation, and nitrosylation (Figure 2A, B). Moreover, phosphorylation, oxidation, and nitrosylation cause loss of calstabin1 from the RyR1 complex [22,23,36] and Sgcb−/− muscle RyR1 were depleted of calstabin1 (Figure 2A, B).

Treatment with the 1,4-benzothiazepine derivative, S107, inhibits calstabin1 depletion from the RyR1 complex, stabilizes the closed state of the RyR1 channel, and improves muscle strength in *mdx* mice as well as in 24-month-old mice with age-related muscle weakness [22,23]. We therefore examined whether S107 could inhibit the loss of muscle function in Sgcb−/− mice by randomizing Sgcb−/− mice to receive drinking water without (*n* = 6) or with S107 (25 mg/100 mL, *n* = 6). The treatment persisted for approximately 4 weeks after which the animals were sacrificed and biochemistry and muscle function were assayed. Immunoprecipitation and immunoblotting of RyR1 indicated that there was increased calstabin1 bound to RyR1 in the S107 treated Sgcb−/− mice (Figure 2A, B).

**Figure 3:**
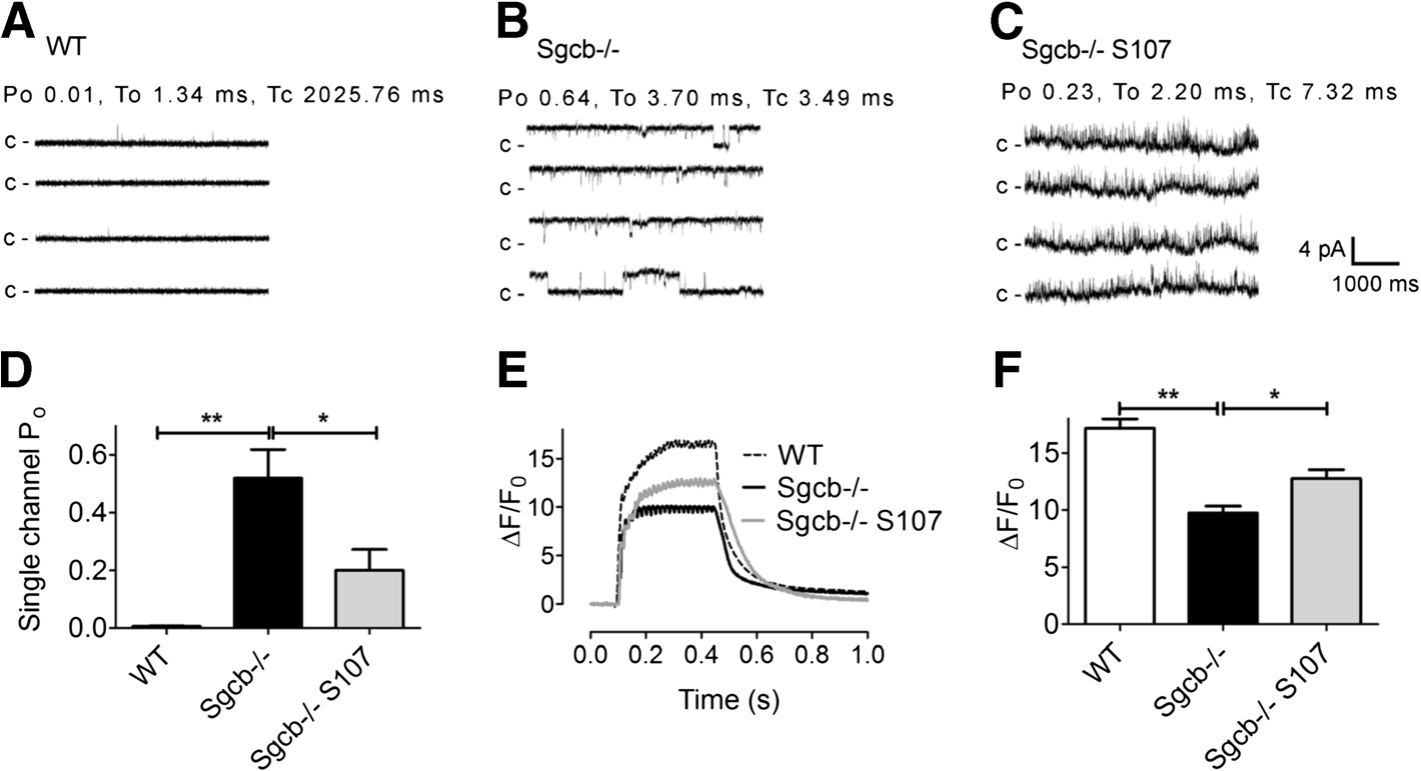
**β-sarcoglycan deficient muscle displays RyR1 dysfunction and defective SR Ca**^**2+**^**release that is restored by S107 treatment.** (**A**-**C**) Representative RyR1 single channel current traces in samples from WT (A), Sgcb−/− (**B**), and Sgcb−/− S107 (C) treated mice. Channel activity was measured at 90 nmol/L (nM) free cytosolic [Ca^2+^]. Channel openings are shown as upward deflections; the closed (c -) state of the channel is indicated by horizontal bars in the beginning of each tracing. For each group, channel activity is illustrated by four different traces, each of 5 s length as indicated by dimension bars. The single channel open probability (Po), To (mean open time) and Tc (mean closed time) were calculated from a 2 min recording under 90 nmol/L free cytosolic [Ca^2+^] are shown above the upper trace. (**D**) Bar graph summarizing RyR1 single channel Po under 90 nmol/L free cytosolic [Ca^2+^] from WT (*n* = 4; white bar), Sgcb−/− (*n* = 3; black bar), and Sgcb−/− + S107 (*n* = 4; red bar) samples. Data presented as mean ± S.E.M; * *P* <0.05; ** *P* <0.01 (ANOVA). (**E**) Representative tetanic Ca^2+^ transients (normalized Fluo-4 fluorescence) in FDB muscle fibers from wild-type (WT), β-sarcoglycan-deficient control (Sgcb−/−), and S107-treated β-sarcoglycan-deficient (Sgcb−/− S107) mice. (**F**) Average Ca^2+ ^transient amplitudes (±SEM, *n* = 6 (WT) *n* =20 (Sgcb−/−), *n* = 26 (Sgcb−/− S107) cells from three mice in each group, * *P* <0.05, ** *P* <0.01 (ANOVA)).

Preserved RyR1-calstabin1 interaction is associated with reduced SR Ca^2+ ^leak, improved Ca^2+ ^release, muscle function, and exercise capacity [18]. To assess the presence of RyR1-dependent Ca^2+ ^leak, we measured single channel open probability (P_o_) of the RyR1 using SR membranes from fast twitch muscles that were fused to planar lipid bilayers. Experimental conditions mimicking resting skeletal muscle (90 nM Ca^2+ ^on the *cis*, ‘cytosolic’ side) were used. The P_o_ of RyR1 from the Sgcb−/− mice was increased (Figure 3A, B, and D), and S107 treatment resulted in a significant reduction in RyR1 P_o_ (Figure 3C, D). These data are consistent with ‘leaky’ RyR1 [22,23]. To study SR Ca^2+ ^release, we loaded isolated fast twitch flexor digitorum brevis (FDB) muscle fibers with the fluorescent Ca^2+ ^indicator Fluo-4 AM and electrically stimulated the fibers to produce tetanic contractions. Ca^2+^ transients were reduced in FDB myocytes from Sgcb−/− mice (Figure 3E, F). The S107-treated Sgcb−/− displayed increased Ca^2+^ transients compared to untreated Sgcb−/− (mean tetanic F/F_0_ ± SEM: WT, 17 ± 0.8 (*n* = 6); Sgcb−/−, 10 ± 0.6 (*n* = 20); Sgcb−/− S107, 13 ± 0.8 (*n* = 26); cells were taken from three mice per group, *P* < 0.05 (ANOVA); Figure 3E, F).

**Figure 4:**
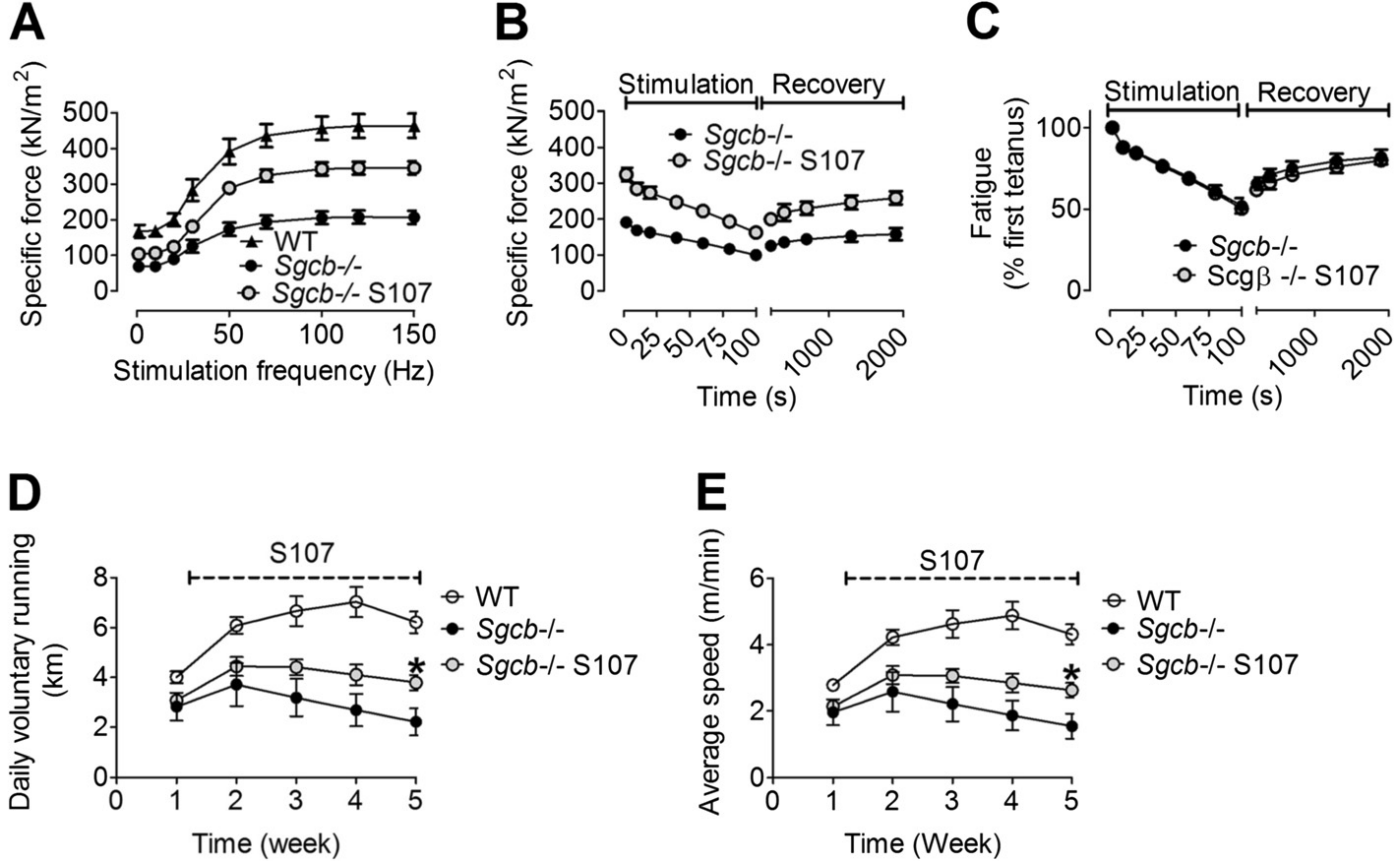
**S107 treatment increases muscle force and exercise capacity in β-sarcoglycan deficient mice.** (**A**) Force-frequency curves of EDL muscle from WT control, β-sarcoglycan-deficient (Sgcb−/−), and S107-treated Sgcb−/− (Sgcb−/− S107) mice. (**B**) Fatigue stimulation (50 tetani; each tetanic stimulation had a duration of 350 ms and was produced by stimulating the muscle with 0.5 ms pulses at 70 Hz frequency) on the same muscles as (A). (**C**) Relative decline in force production during fatigue in (B). EDL force measurements are presented as mean ± SEM, *n* = 6–9. (**D**, **E**) Exercise capacity in Sgcb−/− mice is improved by S107. Daily voluntary running distance (D) and average running speed (E). Pooled data are presented as mean ± SEM, *n* = 8–5, * *P* <0.05 (ANOVA).

We next measured force production in isolated EDL muscles. There was a significant increase in EDL specific force in the S107-treated Sgcb−/− mice (mean tetanic forces at 70 Hz stimulation ± SEM: Sgcb−/− S107, 320 ± 20 kNm^-1^, Sgcb−/− control 200 ± 20 kNm^-1^; *n* = 9 (Sgcb−/−), *n* = 6 (Sgcb−/− S107), *P* <0.001 (*t*-test); Figure 4A). A marked feature of skeletal muscle is its susceptibility to fatigue and recovery. EDL muscles from S107-treated and untreated Sgcb−/− mice were repeatedly stimulated to tetanic contractions. The degree of force reduction during fatigue as well as the recovery was similar in both groups (Figure 4B, C). However, the EDL from S107-treated mice exhibited increased force production prior to the fatigue protocol. Therefore the EDL from S107-treated mice exhibited higher force production throughout the fatigue protocol and would likely sustain higher levels of work *in vivo*[30,37]. To determine whether the improvements in muscle function corresponded to increased exercise capacity, voluntary running performance was recorded in S107-treated and untreated Sgcb−/− mice. S107-treated Sgcb−/− mice ran longer and faster (mean daily running distance after 5 weeks ± SEM: WT, 6.2 ± 0.4 km, Sgcb−/− S107, 3.8 ± 0.3 km, Sgcb−/− 1.5 ± 0.4 km, *n* = 8–5, *P* <0.05 (ANOVA); Figure 4D, E).

In the present study we show that RyR1 in dystrophic muscle are oxidized, cysteine-nitrosylated, phosphorylated, and depleted of calstabin1, resulting in ‘leaky’ channels, decreased fast twitch muscle force, and impaired exercise capacity. Furthermore, we show that treating β-sarcoglycan-deficient mice with the RyR stabilizing drug, S107, preserves RyR1-calstabin1 binding, increases SR Ca^2+^ release, fast twitch muscle force, and improves voluntary exercise capacity.

Mutations in components of the DGC or in DGC-associated proteins cause several different muscular dystrophies, including DMD, the congenital muscular dystrophies, and LGMD [4]. Previous studies have shown that SR Ca^2+^ release is reduced in muscle from the dystrophic *mdx* mouse model [15-17]. Moreover, it was recently reported that mdx muscle display increased Ca^2+^ spark frequency [23,38]. This is consistent with increased RyR1-mediated Ca^2+ ^leak. In the present study, leaky RyR1 was seen in Sgcb−/− muscle as evidenced by increased RyR1 open probability (Figure 3A-D). Interestingly, it was recently shown that overexpression of the SR Ca^2+ ^ATPase (SERCA) in dystrophic mice could rescue the pathological phenotype in the muscle by effectively pumping excess Ca^2+^ back into the SR [33]. Taken together, these data indicate that intracellular Ca^2+ ^leak is a prominent, but reversible, pathological mechanism in muscular dystrophies. It is possible that cessation of Ca^2+^ leak would lead to reduction of diverse pathogenic signals in muscular dystrophy, including those affecting gene expression, protease activity, or redox homeostasis. For instance, the activity of Ca^2+^-dependent proteases such as the calpains are increased in muscular dystrophy and have been attributed a role in the breakdown of myofillament proteins [33,39]. Inhibition of this process has been suggested as a therapeutic strategy in myopathies [8]. In addition to improving SR Ca^2+ ^release, S107 treatment could potentially lead to increased muscle force by preventing Ca^2+^-dependent remodeling of the myofilaments.

Electron micrographs from Sgcb−/− EDL muscles displayed abnormal mitochondrial morphology (Figure 1F, G). Mitochondrial defects have previously been described in both patients [34] and murine models [9,31] of muscular dystrophy. Ultrastructural analysis of diaphragm muscle from α-sarcoglycan-null mice revealed disrupted and swollen mitochondria [31]. Furthermore, Ca^2+^ overload leading to mitochondrial dysfunction has been linked to activation of cell death pathways in δ-sarcoglycan deficient mice [9], and we have recently reported that mitochondrial ROS dependent oxidation of RyR1 creates a vicious cycle of SR Ca^2+^ leak via RyR1 causing mitochondrial Ca^2+^ overload and exacerbating mitochondrial ROS production in muscle aging [22].

Cardiomyopathy is a common symptom of muscle dystrophy [40,41] and improved cardiac function is seen following S107 treatment of heart failure (post-myocardial infarction) and in *mdx* mice [41,42]. Sgcb−/− mice that were treated with S107 displayed improved exercise capacity, measured as voluntary running distance and speed. Exercise capacity is a compound measure that involves the function of several organ systems. Therefore, it is possible that improved cardiac function in Sgcb−/− mice following S107 treatment could contribute to the improved running capacity, this is unlikely however since the cardiac function was normal by echocardiography in these mice (data not shown). Moreover, muscle function is a central determinant of exercise capacity [37] and the reduced tetanic Ca^2+ ^and impaired muscle specific force that is seen in Sgcb−/− were improved by fixing the skeletal muscle SR Ca^2+ ^leak with S107 and these features were associated with improved voluntary exercise.

## Conclusions

We show here that remodeling of the RyR1 contributes to skeletal muscle weakness and reduced exercise capacity in Sgcb−/− mice, a model of LGMD. This is consistent with results from a previous study of the *mdx* mouse, in which RyR1 were S-nitrosylated, and displayed SR Ca^2+ ^leak through the RyR1 [23]. The pathophysiological similarities between the two types of muscular dystrophy, which both result from disruption of the DGC, suggest that RyR1-mediated SR Ca^2+^ leak is a common mechanism for DGC-related muscular dystrophy. Furthermore, this mechanism can be targeted for treatment with the orally available 1,4-benzothiazepine derivative S107. Thus, the present findings suggest the possibility of a novel therapeutic strategy in muscular dystrophy.

## Abbreviations

DGC: Dystrophin-glycoprotein complex; DMD: Duchenne muscular dystrophy; EDL: Extensor digitorum longus; LGMD: Limb-girdle muscular dystrophy; RyR1: Ryanodine receptor; Sgcb−/−: β-Sarcoglycan deficient mice; SR: Sarcoplasmic reticulum.

## Competing interests

ARM is a consultant for a start-up company, ARMGO Pharma Inc., which is targeting RyR1 to improve exercise capacity in muscle diseases.

## Authors’ contributions

DCA designed experiments, conducted experiments, analyzed data, and wrote the first draft of the paper. ACM conducted single channel studies. SR did the biochemistry. MJB performed calcium measurements. AU did muscle function studies. TS did the pathology. JD helped design experiments and analyze data. ARM conceived of the study, designed the experiments, analyzed data, and revised the manuscript. All authors read and approved the final manuscript.
